# CD200Fc reduces LPS-induced IL-1β activation in human cervical cancer cells by modulating TLR4-NF-κB and NLRP3 inflammasome pathway

**DOI:** 10.18632/oncotarget.16596

**Published:** 2017-03-27

**Authors:** Aiqin He, Jia Shao, Yu Zhang, Hong Lu, Zhijun Wu, Yunzhao Xu

**Affiliations:** ^1^ Department of Gynecology Oncology, Nantong Tumor Hospital, Tumor Hospital Affiliated to Nantong University, Nantong, China; ^2^ Department of Obstetrics and Gynecology, Affiliated Hospital of Nantong University, Nantong, China

**Keywords:** CD200Fc, cervical cancer, TLR4-NF-κB, NLRP3 inflammasome, IL-1β

## Abstract

Chronic inflammation plays an important role in tumorigenesis of cervical cancer. CD200Fc, a CD200R1 agonist, has been found to have anti-inflammatory effects in autoimmune diseases and neuro-degeneration. However, the anti-inflammatory effect of CD200Fc on cervical cancer has not yet to be completely understood. This study investigated the anti-inflammatory effects and mechanisms of CD200Fc in LPS-induced human SiHa cells and Caski cells. SiHa cells and Caski cells were stimulated with 40 μg/ml LPS under different concentrations of CD200Fc for 90 min or 12 hours. The mRNA and protein levels of pro-IL-1β, cleaved-IL-1β and NLRP3, as well as the protein level of cleaved caspase-1, were significantly increased in LPS-induced SiHa cells and Caski cells. LPS stimulation did not change ASC and pro-caspase-1 expression. CD200Fc down-regulated protein expression of cleaved caspase-1 and mRNA and protein expression of pro-IL-1β, cleaved-IL-1β and NLRP3. In addition, the protein levels of TLR4, p-P65 and p-IκB, as well as the translocation of P65 to nucleus, were significantly increased in LPS-induced SiHa cells and Caski cells. LPS stimulation did not change t-P65 and t-IκB on protein levels, which were components of TLR-NF-κB pathway. CD200Fc down-regulated protein expression of TLR4, p-P65 and p-IκB and inhibited the translocation of P65 to nucleus in LPS-induced SiHa cells and Caski cells. These results indicated that CD200Fc appeared to suppress the inflammatory activity of TLR4-NF-κB and NLRP3 inflammasome pathway in LPS-induced SiHa cells and Caski cells. It provided novel mechanistic insights into the potential therapeutic uses of CD200Fc for cervical cancer.

## INTRODUCTION

Cervical cancer (CC) is the second most common cancer in females worldwide [1]. Reports have suggested that human papillomavirus (HPV), such as HPV 16 and 18, are well established as an etiological agent for CC and have the ability to transform normal cervical cells into neoplastic cells [2, 3]. However, infection with HPV by itself is thought to be insufficient for the malignant transformation of HPV infected normal cervical cells [4]. Recently, with the deepening research on CC, it has been confirmed that HPV infection along with persistent chronic inflammation can induce carcinogenesis [2, 4].

Toll-like receptors (TLRs) are a system of innate immune defense. It has been well established that HPV induce TLR4 expression and interfere in TLR4-NF-κB pathways, leading to persistent chronic inflammation and carcinogenesis [5–7]. Our recent study showed that LPS induced the activation of TLR4-NF-κB pathway in CC SiHa (HPV16+) cells, but not in HeLa (HPV18+) and C33A (HPV-) cells [5]. Increasing evidences have shown that resistance to chemotherapy is strongly dependent on the HPV infection and the activation of TLR4-NF-κB pathways in CC [6, 8, 9]. The TLR4-NF-κB pathway and its regulation are highly complicated, and may lead to cancer invasion and chemoresistance in multiple ways, including modulation of the tumoural microenvironment through the production of inflammatory mediators, such as IL-1β [8–10].

In recent years, the role of NOD-like receptor family, pyrin domain containing 3 (NLRP3) inflammasome in cancer is now attracting widespread attention [11]. The NLRP3 inflammasome is tightly controlled by NF-κB [12, 13]. The NLRP3 inflammasome complex mediate the maturation of inactive pro-caspase-1 into active cleaved-caspase-1, and promote the maturation of pro-inflammatory cytokines IL-1β from pro-IL-1β to ignite inflammation [14, 15]. However, whether HPV infection and chronic inflammation in CC require the activation of NLRP3 inflammasome remains unclear.

CD200, a membrane glycoprotein of the immune-globulin superfamily, have been shown strong effect on immune suppression via the interaction with its receptor CD200R [16, 17]. Experimental studies have demonstrated that CD200-CD200R axis plays a key role in the modulation of inflammatory responses in autoimmune diseases and neuro-degeneration [17, 18]. Soluble CD200 fusion protein (CD200Fc) is a CD200 fusion protein consisting of the extracellular domain of CD200 bound to a murine IgG2aFc sequence and modified to eliminate Fc receptor and complement binding regions [19, 20]. Recent reports have showed that the effects of CD200Fc in attenuating the release of pro-inflammatory cytokines and glial cell activation [21] in neuro-inflammatory diseases [20, 22, 23]. Nevertheless, recent knowledge remains limited as to the anti-inflammatory effects of CD200Fc in LPS-induced SiHa cells and Caski cells.

Therefore, the goal of our present study was to explore whether CD200Fc might regulate LPS-induced IL-1β activation in human CC SiHa cells and Caski cells. Furthermore, efforts have been taken to identify the TLR4-NF-κB and NLRP3 inflammasome pathways underlying the anti-inflammatory effect of CD200Fc.

## RESULTS

### CD200Fc down-regulated the production and activation of IL-1β in LPS-stimulated SiHa cells and Caski cells

**Figure 1:**
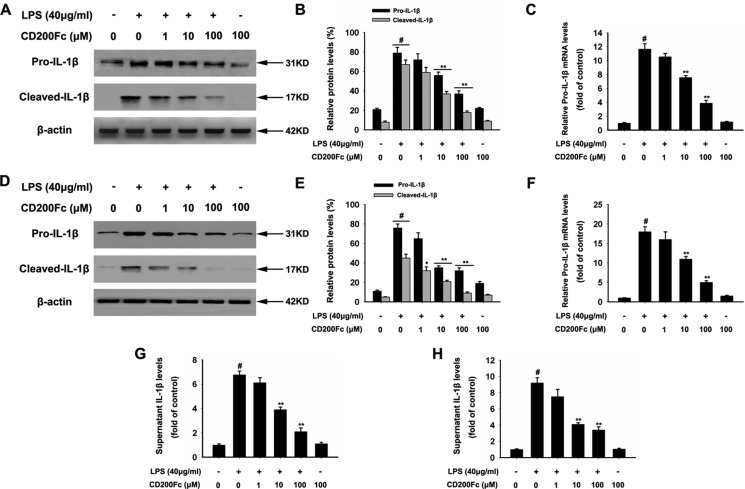
Effects of CD200Fc on production and activation of IL-1β in LPS-stimulated SiHa cells and Caski cells SiHa cells and Caski cells were stimulated with 40 μg/ml LPS under different concentrations of CD200Fc for 12 hours. The protein levels of pro-IL-1β and cleaved-IL-1β were measured by western blot analysis in SiHa cells (**A**) and Caski cells (**D**). The bar chart showed the ratio of pro-IL-1β and cleaved-IL-1β to β-actin at each groups in SiHa cells (**B**) and Caski cells (**E**). The mRNA level of pro-IL-1β was measured by qRT-PCR analysis. The bar chart showed the ratio of pro-IL-1β to β-actin at each groups in SiHa cells (**C**) and Caski cells (**F**). The extracellular levels of I IL-1β in culture media were measured using commercial ELISA kits in SiHa cells (**G**) and Caski cells (**H**). Data are the mean ± S.E.M. of three independent experiments. ^#^*P* < 0.001 vs. control group (cultured in medium alone); **p < 0.001 vs. LPS-induced group.

Firstly, the effects of CD200Fc on the production and activation of IL-1β from LPS-simulated SiHa cells and Caski cells were evaluated. Western blot results showed that LPS stimulation significantly enhanced the pro-IL-1β and cleaved IL-1β level relative to that observed in the un-stimulated cells (Figure 1A–1B and 1D−1E). The production of pro-IL-1β and cleaved IL-1β in response to LPS was significantly inhibited by CD200Fc in a dose-dependent manner. In addition, qRT-PCR results (Figure 1C and 1F) and ELISA analysis (Figure 1G and 1H) showed that CD200Fc inhibited the secretion of pro-IL-1β after LPS exposure. However, no apparent change of IL-1β production and activation was observed between control and 100 μM CD200Fc treatment in normal conditions.

### CD200Fc inhibited the expression of NLRP3 inflammasome components in LPS-stimulated SiHa cells and Caski cells

**Figure 2:**
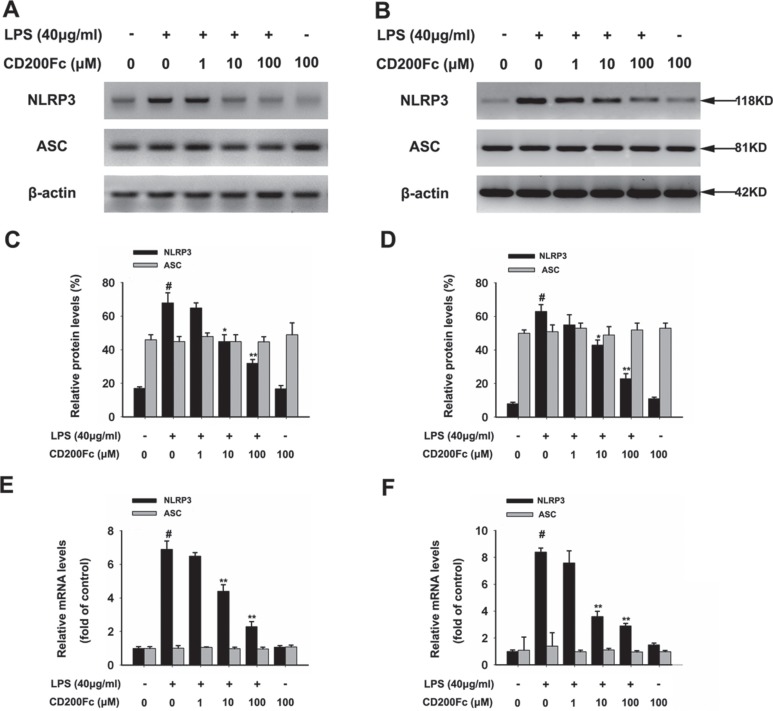
Effects of CD200Fc on the expression of NLRP3 inflammasome components in LPS-stimulated SiHa cells and Caski cells SiHa cells and Caski cells were stimulated with 40 μg/ml LPS under different concentrations of CD200Fc for 90 min. The protein levels of NLRP3 and ASC were measured by western blot analysis in SiHa cells (**A**) and Caski cells (**B**). The bar chart showed the ratio of NLRP3 and ASC to β-actin at each groups in SiHa cells (**C**) and Caski cells (**D**). The mRNA levels of NLRP3 and ASC were measured by qRT-PCR analysis. The bar chart showed the ratio of NLRP3 and ASC to β-actin at each groups in SiHa cells (**E**) and Caski cells (**F**). Data are the mean ± S.E.M. of three independent experiments. ^#^*P* < 0.001 vs. control group (cultured in medium alone); *p < 0.01, **p < 0.001 vs. LPS-induced group.

The NLRP3 inflammasome components, such as NLRP3 and ASC, are the initiators of inflammatory responses[11]. Western blot results showed that the protein expression of NLRP3 in SiHa cells and Caski cells was significantly increased 3 hours after LPS stimulation (Figure 2A–2D). The addition of CD200Fc to the cells reduced the protein expression of NLRP3. In addition, qRT-PCR results showed that incubation with CD200Fc dose-dependently inhibited this LPS-induced mRNA expression of NLRP3 (Figure 2E–2F). However, no apparent protein and mRNA change of ASC was observed in LPS and/or CD200Fc treatment group (Figure 2A–2F).

### CD200Fc inhibited cleaved caspase-1 production in LPS-stimulated SiHa cells and Caski cells

**Figure 3:**
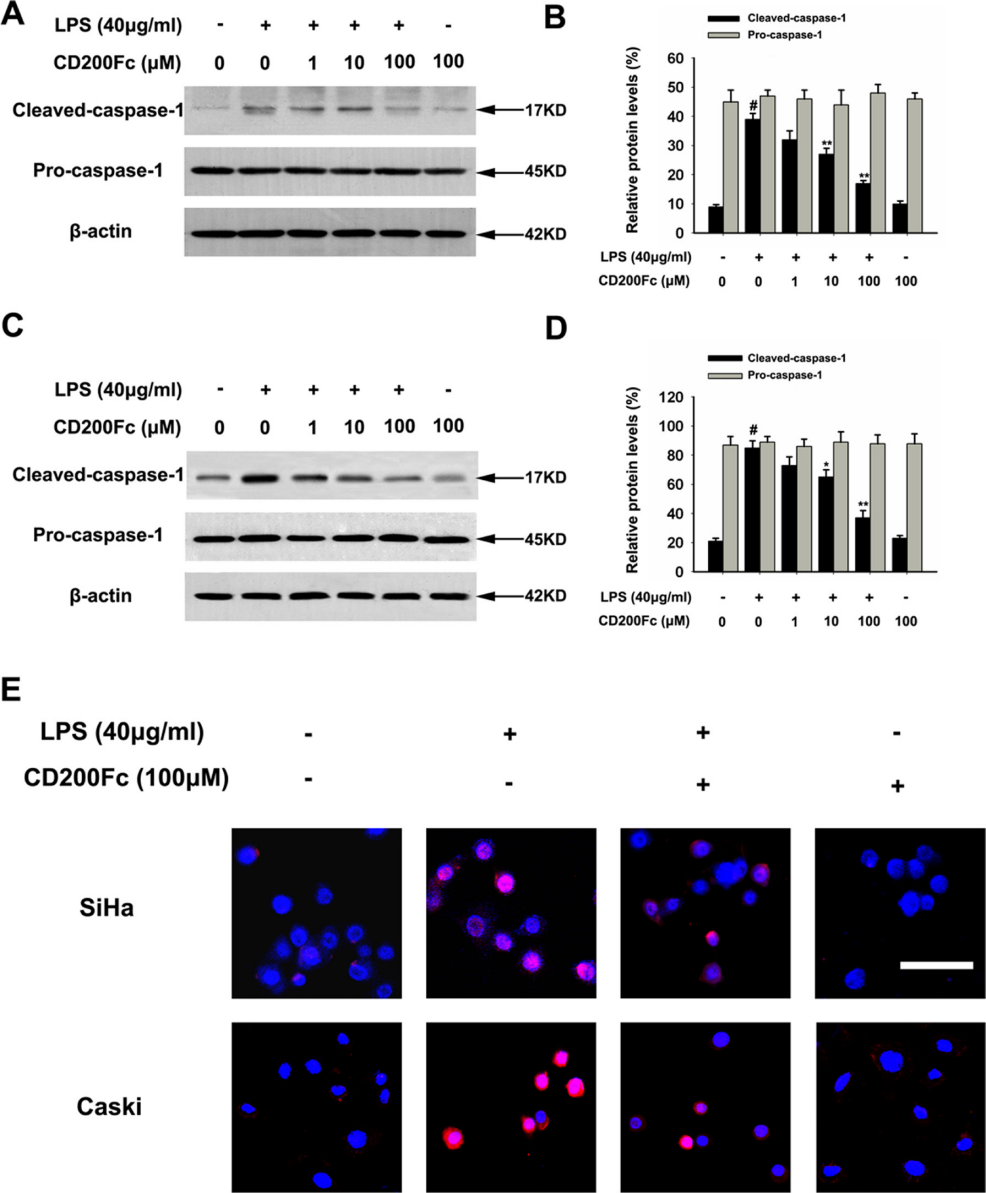
Effects of CD200Fc on cleaved caspase-1 production in LPS-stimulated SiHa cells and Caski cells SiHa cells and Caski cells were stimulated with 40 μg/ml LPS under different concentrations of CD200Fc for 12 hours. The protein levels of cleaved-caspase-1 and pro-caspase-1 were measured by western blot analysis in SiHa cells (**A**) and Caski cells (**C**). The bar chart showed the ratio of cleaved-caspase-1 and pro-caspase-1 to β-actin at each groups in SiHa cells (**B**) and Caski cells (**D**). The expression level of cleaved-caspase-1 in SiHa cells and Caski cells was measured by immunofluorescent staining (**E**). Meanwhile, the phenotype of nuclei was also investigated via DAPI staining. Scale Bar = 50 μm. Data are the mean ± S.E.M. of three independent experiments. ^#^*P* < 0.001 vs. control group (cultured in medium alone); *p < 0.01, **p < 0.001 vs. LPS-induced group.

Caspase-1 is a member of a family of caspases with large prodomains, and its activation is required to cleave pro-IL-1β into IL-1β [15]. Therefore, western blot analysis and immunofluorescent staining were used to determine whether CD200Fc treatment affected the cleavage of caspase-1 in LPS-stimulated SiHa cells and Caski cells. As shown in Figure 3, LPS increased the cleavage of caspase-1, while treatment with various doses of CD200Fc reduced the cleaved forms of caspase-1 in SiHa cells and Caski cells. In addition, no apparent protein change of pro-caspase-1 was observed in LPS and/or CD200Fc treatment group (Figure 3). These results suggested involvement of the NLRP3 inflammasome during CD200Fc mediated anti-inflammatory effects in LPS-stimulated SiHa cells and Caski cells.

### CD200Fc reduced the activation TLR4-NF-κB pathways in LPS-stimulated SiHa cells and Caski cells

**Figure 4:**
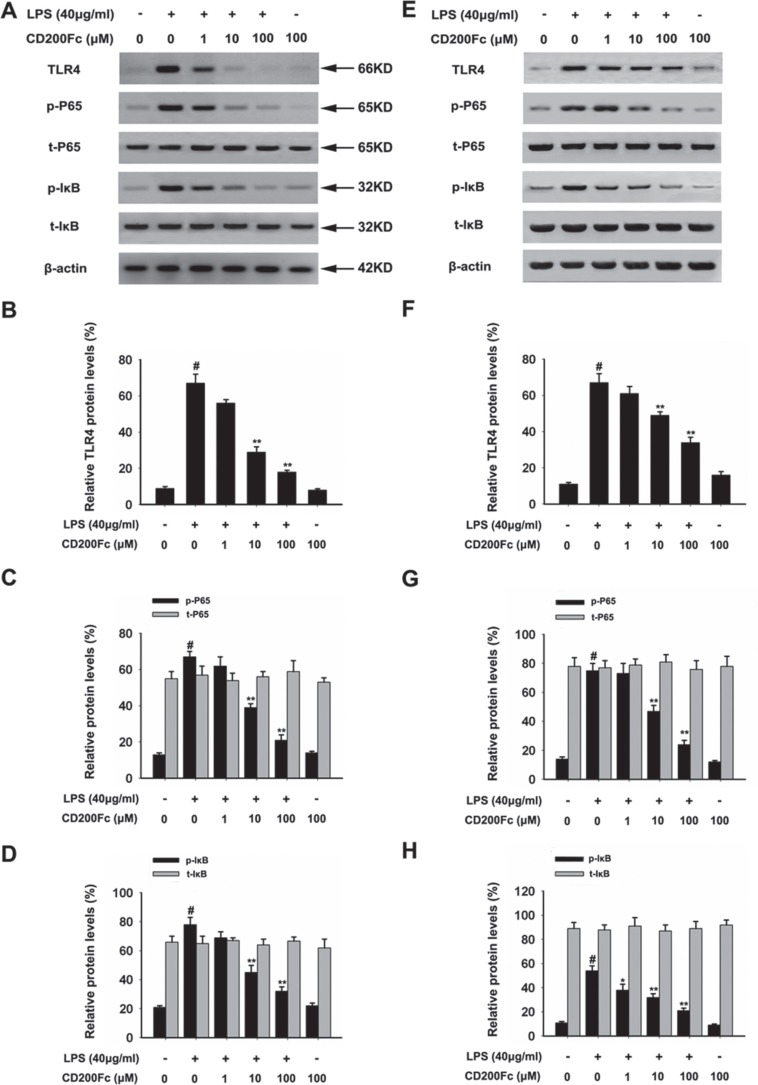
Effects of CD200Fc on the protein levels of TLR4, p-P65, t-P65, p-I-κB and t-I-κB in LPS-induced SiHa cells and Caski cells SiHa cells and Caski cells were stimulated with 40 μg/ml LPS under different concentrations of CD200Fc for 90 min. The protein levels of TLR4, p-P65, t-P65, p-I-κB and t-I-κB were measured by western blot analysis in SiHa cells (**A**) and Caski cells (**E**). The bar chart showed the ratio of TLR4 to β-actin at each groups in SiHa cells (**B**) and Caski cells (**F**). The bar chart showed the ratio of p-P65 and t-P65 to β-actin at each groups in SiHa cells (**C**) and Caski cells (**G**). The bar chart showed the ratio of p-I-κB and t-I-κB to β-actin at each groups in SiHa cells (**D**) and Caski cells (**H**). Data are the mean ± S.E.M. of three independent experiments. ^#^*P* < 0.001 vs. control group (cultured in medium alone); *p < 0.01, **p < 0.001 vs. LPS-induced group.

TLR4 is a key regulators involved in regulating the LPS-induced inflammatory mediators expression through the activation of NF-κB pathway [8]. As shown in Figure 4A–4B and 4E−4F, treatment with CD200Fc reduced the protein expression of TLR4, which was induced by LPS for 90 min in SiHa cells and Caski cells.

**Figure 5:**
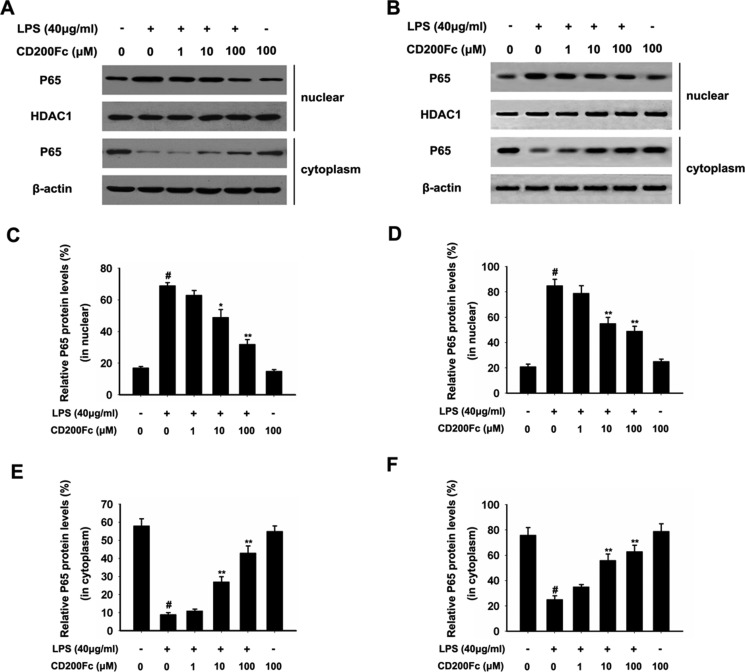
Effects of CD200Fc on the translocation of P65 from cytoplasm to nucleus in LPS-induced SiHa cells and Caski cells SiHa cells and Caski cells were stimulated with 40 μg/ml LPS under different concentrations of CD200Fc for 90 min. Nuclear and cytosolic extracts were isolated and the levels of P65 in each fraction were determined by western blot analysis in SiHa cells (**A**) and Caski cells (**B**). HDAC1 and β-actin were used as internal controls. The bar chart showed the ratio of P65 to HDAC1 in nuclear at each groups in SiHa cells (**C**) and Caski cells (**D**). The bar chart showed the ratio of P65 to β-actin in cytosolic at each groups in SiHa cells (**E**) and Caski cells (**F**). Data are the mean ± S.E.M. of three independent experiments. ^#^*P* < 0.001 vs. control group (cultured in medium alone); *p < 0.01, **p < 0.001 vs. LPS-induced group.

To further investigate the activation of NF-κB pathways involved in the mechanism in LPS-induced SiHa cells and Caski cells, we assessed the components the NF-κB pathway (P65 and I-κB) in SiHa cells and Caski cells. As shown in Figure 4A, 4C–4E and 4G–4H, LPS treated for 90 min strongly induced phosphorylation of P65 and I-κB. Treatment with CD200Fc reduced expressions of p-P65 and p-I-κB in LPS-induced SiHa cells and Caski cells. However, no apparent protein change of t-P65 and t-I-κB was observed between control and CD200Fc treatment. In addition, western blot data showed a marked increased translocation of P65 from cytoplasm to nucleus after exposure to LPS. However, LPS-induced P65 level in the nuclear fractions was reduced by CD200Fc treatment (Figure 5A–5C). Taken together, the above findings demonstrated involvement of the TLR4-NF-κB pathway in the anti-inflammatory effect of CD200Fc in LPS-induced SiHa cells and Caski cells.

## DISCUSSION

The model of immunological stress provided by LPS *in vitro* or *in vivo* is generally used to increase the release of excessive inflammatory mediators, such as IL-1β, and also causes the tumorigenesis of CC [5, 24]. Our previous study have shown that LPS stimulation activates the TLR4-NF-κB pathway in SiHa cells by acting on TLR4 to activate NF-κB, and consequently activates the expression of inflammatory mediators, such as IL-1β [5]. However, almost no information is available concerning whether LPS stimulation could activates the NLRP3 inflammasome pathway in SiHa cells and Caski cells. The present study firstly showed that LPS stimulation enhanced the mRNA and protein expression of NLRP3 inflammasome components NLRP3 and cleaved-caspase-1 in SiHa cells and Caski cells, which illustrated that NLRP3 inflammasome pathway might also play a role in the tumorigenesis of CC.

CD200-CD200R axis is a regulatory system of inflammation that plays a critical role in various diseases, a strengthened inflammatory response was usually observed when the CD200-CD200R conjugation was impaired [16, 17]. In contrast, enhanced expression of CD200R by CD200Fc treatment alleviates pathological effects of inflammation [16, 17, 21, 23, 25]. However, the anti-inflammatory effects of CD200Fc in CC have not heretofore been investigated. The present study provided the first evidence that CD200Fc attenuated LPS-induced production and activation of IL-1β, as well as mechanical inhibition of TLR-NF-κB and NLRP3 inflammasome pathways.

IL-1β is considered to be an endogenous pyrogen and also plays a vital role in promoting a variety of innate immune processes associated with infection, inflammation and autoimmunity [14, 15]. It appears that IL-1β can contribute to the pathogenesis of HPV-infected cervical carcinoma. Recent studies have shown that IL-1β is associated with development of cervical carcinoma with persistent HPV16/18 infection [26]. Recently, a meta-analysis suggested that the IL-1β polymorphisms may contribute to genetic susceptibility of CC [27, 28]. In addition, Niebler et al. found that attenuation of IL-1β by the HPV16 E6 oncoprotein in HPV-positive cervical carcinoma immortalized cells is apparently a crucial step in viral immune evasion and initiation of malignancy of CC [29]. Moreover, recent studies showed that CD200Fc suppressed the LPS-induced release of IL-1β in rat primary microglial cells [21] and human renal proximal tubular epithelial cells [23]. The present study showed, for the first time, that the addition of CD200Fc could significantly reduce the LPS-induced production and activation of pro-IL-1β and cleaved-IL-1β in SiHa cells and Caski cells.

The generation of pro-IL-1β and cleaved (mature)-IL-1β is tightly controlled by the NLRP3 inflammasome, which has been intensively studied [15, 30]. Upon sensing danger signals such as LPS, NLRP3 proteins oligomerize and recruit caspase-1 through ASC [15]. Subsequently, pro-caspase-1 undergoes an autocatalytic activation. Finally, mature caspase-1 cleaves pro-IL-1β to produce cleaved-IL-1β [14, 30, 31]. Pontillo et al. found that NLRP3 inflammasome pathway could affect HPV virus/persistence and cervical cancer progression [32]. Moreover, the findings of Abdul-Sater et al. demonstrated that NLRP3-dependent caspase-1 activation in cervical epithelial cells contributes to development of the chlamydial inclusion and cervical cancer progression [33]. For the first time, our findings demonstrated that the addition of CD200Fc could inhibit the mRNA and protein expression of NLRP3, and reduce cleaved caspase-1 production in a dose-dependent manner. This result indicated that the regulatory effects of CD200Fc on the activation of pro-IL-1β and cleaved-IL-1β might be partly attributable to the regulation of caspase-1 activation via NLRP3 inflammasome pathway in SiHa cells and Caski cells.

The NLRP3 inflammasome is tightly controlled by the TLR4-NF-κB signaling pathway [31]. LPS can activate downstream of the TLR-NF-κB signaling pathway [8, 9]. The activation of NF-κB leads to activation of the NLRP3 inflammasome and cytokine secretion, which plays an important role in cervical cancer progression [8, 9, 34]. In addition, recent studies showed that CD200Fc suppressed the TLR4-NF-κB signaling pathway-mediated release of IL-1β in LPS-induced rat primary microglial cells [21] and human renal proximal tubular epithelial cells [23]. The present study showed that LPS stimulation significantly enhanced the TLR4 expression, the phosphorylation of P65 and I-κB, and increased P65 level in the nuclear fractions, indicating that LPS promoted activation of TLR4-NF-κB pathway in SiHa cells and Caski cells. At the same time, CD200Fc treatment could significantly inhibit LPS-induced increase in TLR4 expression, the phosphorylation of P65 and I-κB, and P65 level in the nuclear fractions. We postulated that the anti-inflammatory effects of CD200Fc in SiHa cells and Caski cells were mediated by inhibition of TLR4-NF-κB signaling pathway.

**Figure 6:**
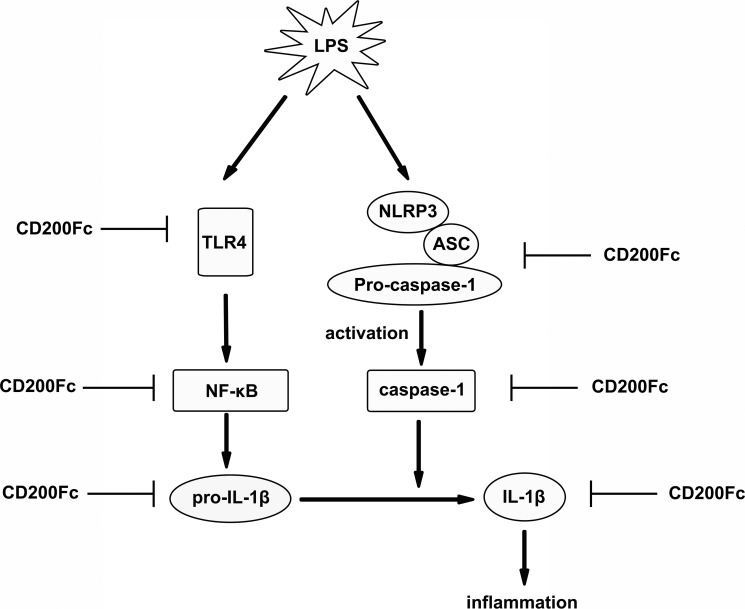
Possible mechanisms by which CD200Fc inhibited production and activation of IL-1β in LPS-stimulated SiHa cells and Caski cells LPS-stimulated activation of TLR4-NF-κB and NLRP3 inflammasome pathways were inhibited by down-regulation of TLR4, p-P65, and p-I-κB, and NLRP3, cleaved caspase-1 after CD200Fc treatment, and subsequent inhibited production and activation of IL-1β.

In conclusion, CD200Fc could inhibit LPS-induced production and activation of pro-IL-1β and cleaved-IL-1β. These effects might appear to be produced by the inhibition of LPS-induced activation TLR4-NF-κB pathway, the mRNA and protein expression of NLRP3, and lead to suppression of the activation of caspase-1 in SiHa cells and Caski cells (Figure 6). The anti-inflammatory activities of CD200Fc in SiHa cells and Caski cells may be attributed to the regulation of the TLR4-NF-κB and NLRP3 inflammasome pathways. It may be possible that the drugs act on one or all of the above process. Further studies will be carried out to determine the likely mechanism.

## MATERIALS AND METHODS

### Cell culture and treatment

SiHa cells and Caski cells were purchased from Chinese Academy of Sciences and cultured in DMEM medium (Sigma, USA) supplemented with 10% fetal bovine serum (Gibco, USA), 100 units/mL penicillin and 100 μg/mL streptomycin (Sigma, USA). Cells were incubated in a humidified atmosphere with 5% CO2 at 37°C. Cells were seeded in 6-well plates (3 × 10^5^ cells/well) and grew overnight to approximately 70% confluence and followed by stimulating with 40 μg/ml LPS under different concentrations (1, 10, 100 μM) of CD200Fc for 90 min or 12 hours. Untreated medium was used as a negative control in this study. CD200Fc was provided by Genentech Inc. (San Francisco, USA).

### Western blot

After washed with pre-chilled phosphate buffered solution (PBS), SiHa cells and Caski cells were harvested and lysed in cell lysate buffer (1 × RIPA, Cell Signaling Technology, USA) for 30 min and centrifuged at 12,000 g for 15 min at 4°C to collect the supernatant. The protein concentration was determined by Bradford Assay (Bio-Rad, USA) and 30 μg of protein was subjected to SDS-polyacrylamide gel electrophoresis (PAGE) and then transferred to a polyvinylidene difluoride membrane (Millipore, USA). After blocking with 5% nonfat milk containing 0.1% Tween 20 at room temperature for 2 hours, the membranes were incubated with the primary antibody at 4°C overnight. The primary antibodies against cleaved IL-1β (1:500), pro-IL-1β (1:800), cleaved-caspase-1 (1:300), pro-caspase-1 (1:500), ASC (1:800) were from Cell Signaling Technology (Danvers, MA). Primary antibody against NLRP3 (1:1,000) was from Novus Biologicals (Littleton, CO). Primary antibodies against TLR4 (1:1,000), p-P65 (1:600), t-P65 (1:600), p-IκB (1:500), or t-IκB (1:500) were from Sigma (St Louis, MO). Primary antibodies against HDAC1 (1:800) and β-actin (1:1,000) were purchased from Santa Cruz Biotechnology (Inc. USA). After incubating with horseradish peroxidase-conjugated secondary antibody (1:5000), protein was visualized using an enhanced chemiluminescence reagent (Thermo Pierce, USA). The levels of target protein bands were densitometrically determined using Quantity Ones 4.4.1 (Bio-Rad Laboratories, Berkeley, CA). The variation in the density of bands was expressed as fold changes compared with the control in the blot after normalized to β-actin.

### ELISA analysis

IL-1β production in SiHa cells and Caski cells was measured by a commercially available ELISA kit (R&D System, Minneapolis, MN), according to the manufacturer's instructions. Briefly, 200 μL of supernatant was added to each well and incubated at room temperature for 2 hours. All the liquid in the well was removed and the plate was washed three times, 200 μL of conjugate was added to each well and incubated at room temperature for 1 hour. After washing three times, 200 μL substrate solution was added in to each well and incubated for another 20 min in the dark. The absorbance was read at 450 nm within 30 min after 50 μL of stop solution was added. The concentrations of IL-1β in the supernatants were calculated from a standard curve.

### RNA extraction and quantitative real time- (qRT-) PCR

Total mRNA was extracted from SiHa cells and Caski cells using the Trizol reagent (Life Technologies), and reverse transcripted with reverse transcription Kit (Takara Biotechnology Co., Ltd., Dalian, China) according to the manufacturer's instructions. Briefly, amplifications of 50 ng cDNA were performed with an ABI7900HT machine (Applied Biosystems, Carlsbad, CA, USA) in 10 μl reaction mixtures containing 1× TaqMan Universal PCR Master Mix (Applied Biosystems), 200 nM of primers, and 0.125 μl of dual-labeled UPL probe (Roche Applied Science, Basel, Switzerland). The cycling programs were as follows: 95°C for 10 min, followed by 40 cycles of 95°C for 15 s and 60°C for 1 min. The fluorescence signal was detected during the extension step in each cycle. Normalized to β-actin, 22DDCT method was used in the calculation of target gene expression. The primers are as follows: 5′- AGG CTG CTC TGG GAT TC -3′ (forward) and 5′- GCC ACA ACA ACT GAC GC -3′ (reverse) for pro-IL-1β; 5′- GAT CTT CGC TGC GAT CAA CAG-3′ (forward) and 5′- CGT GCA TTA TCT GAA CCC CAC-3′ (reverse) for NLRP3; 5′- TGG GCC TGC AGG AGA TG -3′ (forward) and 5′- ATT TGG TGG GAT TGC CAG -3′ (reverse) for ASC; 5′- GTC GAC AAC GGC TCC GGC -3′ (forward) and 5′- GGT GTG GTG CCA GAT TTT CT′ (reverse) for β-actin. Primers were synthesized by Sangon Biotech (Shanghai, China). The relative of expression levels of IL-1β, NLRP3 and ASC were determined using the 2-ΔΔCt method and shown as fold change over controls. The specificity of amplification was assessed by melting curve analysis and gel electrophoresis.

### Immunofluorescent staining

After stimulation, SiHa cells and Caski cells was washed with PBS and fixed by 4% paraformaldehyde at room temperature for 1 hour. After washing, cells were blocked with 3% bovine serum albumin (BSA) containing 0.1% Triton X-100, 0.05% Tween-20 and 10% donkey serum for 2 hours at room temperature to avoid unspecific staining. Then, the cells were incubated with the primary antibody for cleaved- caspase-1 (1:100) for 24 hours at 4°C. After washing by PBS for three times (5 min for each), TRITC-conjugated secondary antibody and DAPI was added to cells and incubated for 2 hours at room temperature in the dark. After washing with PBS, the cells were analyzed with fluorescence microscopy (Leica, Germany).

### Nuclear and cytoplasmic protein extraction

Cytoplasmic and nuclear protein of SiHa cells and Caski cells were extracted using Nuclear and Cytoplasmic Extraction Reagents purchased from Thermo Fisher strictly according to the manufacturer's instructions. Briefly, cells were harvested and washed with PBS after treatment, and 100 μL of ice-cold CER I was added in to fully suspend the cell pellet. After 10 min incubation on ice, 5.5 μL of ice-cold CER II was added to the tube and vortexed for 5 seconds on the highest setting and vortexed for another 5 seconds after 1 min incubation on ice. The tube was centrifuged at maximum speed in a micro centrifuge (16,000 × g) for 5 minutes at 4°C and the supernatant (cytoplasmic extract) was moved to a clean pre-chilled tube and stored at −80°C until use. The insoluble (pellet) fraction which contains nuclei was suspended by 100 μL of NER and incubated on ice, vortexed on the highest setting for 15 seconds every 10 min, for a total of 40 min. Then, the tube was centrifuged at maximum speed in a micro-centrifuge for 10 min at 4°C and the supernatant (Nuclear extract) was collected to a clean pre-chilled tube and stored at −80°C until use. The distribution of P65 in cytoplasm and nuclear was further determined by western blot.

### Data analysis

Data represent the mean and standard error of the mean (S.E.M). Student's *t-test* was performed for all statistical significance analysis using GraphPad Prism software (Version 5, GraphPad Software, Inc., La Jolla, CA). *P* values < 0.05 was considered statistically significant.
